# Genome-Based Identification of Active Prophage Regions by Next Generation Sequencing in *Bacillus licheniformis* DSM13

**DOI:** 10.1371/journal.pone.0120759

**Published:** 2015-03-26

**Authors:** Robert Hertel, David Pintor Rodríguez, Jacqueline Hollensteiner, Sascha Dietrich, Andreas Leimbach, Michael Hoppert, Heiko Liesegang, Sonja Volland

**Affiliations:** Georg-August University Göttingen, Institute of Microbiology and Genetics, Department of Genomic and Applied Microbiology, Göttingen, Germany; ContraFect Corporation, UNITED STATES

## Abstract

Prophages are viruses, which have integrated their genomes into the genome of a bacterial host. The status of the prophage genome can vary from fully intact with the potential to form infective particles to a remnant state where only a few phage genes persist. Prophages have impact on the properties of their host and are therefore of great interest for genomic research and strain design. Here we present a genome- and next generation sequencing (NGS)-based approach for identification and activity evaluation of prophage regions. Seven prophage or prophage-like regions were identified in the genome of *Bacillus licheniformis* DSM13. Six of these regions show similarity to members of the *Siphoviridae* phage family. The remaining region encodes the *B*. *licheniformis* orthologue of the PBSX prophage from *Bacillus subtilis*. Analysis of isolated phage particles (induced by mitomycin C) from the wild-type strain and prophage deletion mutant strains revealed activity of the prophage regions BLi_Pp2 (PBSX-like), BLi_Pp3 and BLi_Pp6. In contrast to BLi_Pp2 and BLi_Pp3, neither phage DNA nor phage particles of BLi_Pp6 could be visualized. However, the ability of prophage BLi_Pp6 to generate particles could be confirmed by sequencing of particle-protected DNA mapping to prophage locus BLi_Pp6. The introduced NGS-based approach allows the investigation of prophage regions and their ability to form particles. Our results show that this approach increases the sensitivity of prophage activity analysis and can complement more conventional approaches such as transmission electron microscopy (TEM).

## Introduction

Phages are bacterial viruses with two replication modes. Lytic phages initiate their reproduction directly after successful DNA injection into the host cell, which leads to prompt cell lysis and phage particle liberation, whereas temperate phages integrate their DNA into the host genome and replicate in conjunction with the host, lacking a direct lethal effect [1]. These so-called prophages can have positive effects for their hosts like protection against related phages, mediation of antibiotic resistance, expanded metabolic capacities, or transfer of virulence factors (reviewed in [2]). Since such cooperation can last for a long time, the host may reduce its prophage to a minimal size retaining only the beneficial genes [1,3]. Under certain conditions prophages can switch back to the lytic life style and kill their hosts [1]. This behavior is observed if the host faces physiological stress, for example in industrial fermentation processes, where it can heavily interfere with the production or lead to a total collapse of the culture with the consequence of economic damage [4,5].

Since the beginning of the genomic era thousands of bacterial genomes have been sequenced including a large number of prophages. An organism can harbor several prophages and their genomes can account for up to 20% of the host genome [1,6]. This genetic prophage pool consists of approximately 23% of all currently known phage genetic material [7]. At the time of this study, the NCBI viral genome page comprised nearly 1000 defined bacteriophage genomes, including 34 *Bacillus* phage genomes.

Our strain of interest, *Bacillus licheniformis* DSM13, has been completely sequenced [8,9] and a recently updated genome annotation is available [10]. Strains of the species *B*. *licheniformis* are extensively used for industrial applications, especially as production platform for enzymes and antibiotics [11]. The lysogeny of the type strain *B*. *licheniformis* DSM13 was investigated on the equivalent strains LMD 50.16 and LMD 75.15 [12]. The experiments identified one type of phage particle for LMD 50.16 and two for LMD 75.15 [13]. The phage identified in both strains was a PBSX ortholog, a prophage which appears in many strains of the *subtilis* group. The additional phage was strain specific [13,14]. However, it is unknown which genomic regions encode the observed phage particles.

To investigate the activity of prophages in the genome of *B*. *licheniformis* DSM13, a combined bioinformatics and next generation sequencing (NGS)-based approach was applied. Prophage regions were predicted by Prophage Finder [15] and manually evaluated and annotated. A phylogenetic classification of the annotated prophage genes was performed based on their homology to known phage genes. And finally, the NGS-based analysis of the phage particle-derived DNA enabled the identification of three active prophage-encoding regions. We assigned the activity of two phage particles to the encoding genomic loci and identified a third new active region. This new method enables fast and precise investigation of putative active prophage regions, and creates a new base for strain evaluation.

## Material and Methods

### Strains and growth conditions

All *Bacillus* strains used in this investigation are listed in Table 1. Cells were grown in LB [16] at 37°C with vigorous shaking if not otherwise stated.

**Table 1:** *Bacillus* strains used in this study.

Strain	Description	Source	Ref.
*B*. *licheniformis* DSM13	Type strain	DSMZ Braunschweig	[8,9]
*B*. *licheniformis* MW3	Derivate of *B*.* licheniformis* DSM13; Δ*hsdR1*, Δ*mcrA*, Δ*hsdR2*	Prof. Dr. Friedhelm Meinhardt, University Münster	[17]
*B*.* licheniformis* ΔPBSX	Derivate of *B*. *licheniformis* MW3; Δ*hsdR1*, Δ*mcrA*, Δ*hsdR2*, ΔBLi_Pp2	Lab strain collection	[18]
*B*.* licheniformis* ΔPBSX-ΔBLi_Pp3	Derivate of *B*.* licheniformis* ΔPBSX; Δ*hsdR1*, Δ*mcrA*, Δ*hsdR2*, ΔBLi_Pp2, ΔBLi_Pp3	This study	

### Prophage identification

For prediction of prophage regions in the genome sequence of *B*. *licheniformis* DSM13, the web-based tool Prophage Finder [15] was used with standard parameters (E-value 0.5, Hits per Prophage 5, Hit Spacing 5500). The results were manually evaluated and putative phage genes were annotated using InterProScan [19]. Variations in the GC-content were identified using Artemis [20]. The genomes of *Bacillus licheniformis* 9945A (CP005965) [21] and *Bacillus subtilis* 168 (NC_000964) [22] were searched for orthologues of the prophage regions using the script rod_finder (https://github.com/aleimba/bac-genomics-scripts). The range of the prophage regions was defined based on annotation and GC-content composition, and refined by a search for insertion repeats flanking the regions. For this purpose 500 bp up- and downstream of the prophage regions were aligned using Clone Manager (Sci-Ed Software, USA) and putative repeats were manually evaluated.

### Deletion of prophage regions

For the construction of the prophage double mutant *B*. *licheniformis* ΔPBSX-ΔBLi_Pp3, the markerless deletion protocol of Rachinger et al. [18] was applied on the prophage single mutant strain *B*. *licheniformis* ΔPBSX [18]. *B*. *licheniformis* ΔPBSX is based on *B*. *licheniformis* MW3, a restriction-modification (RM) system-negative derivative of *B*. *licheniformis* DSM13 [17]. All PCR reactions (50 μl) consisted of 200 μM deoxynucleotides, 100 ng of template DNA, 5 pmol of each primer and 0.5 U Phusion High-Fidelity DNA Polymerase (Thermo Scientific, Darmstadt, Germany). PCR products were purified after gel electrophoresis using a Qiaquick Gel extraction Kit (Qiagen, Hilden, Germany). Sanger sequencing was performed on an ABI3730XL capillary sequencer (BigDye 3.1 chemistry; Applied Biosystems, Darmstadt). The flanking regions of the deletion targets were amplified by PCR. Flank A was constructed using the respective primer pair A1/A2 and flank B using the primer pair B1/B2 (Table 2). Both flanks were fused via SOE-PCR and cloned into the temperature-sensitive vector pKVM2 using *Bam*HI and *Nco*I restriction sites (*Bam*HI, *Nco*I, FastAP and T4-DNA-ligase (Thermo Scientific, Darmstadt, Germany); *Escherichia coli* TOP10 (Invitrogen, Carlsbad, USA)). *Escherichia coli* S17-1 [23] was used for conjugative transfer [23] of the deletion vector. The deletion of the prophage region BLi_Pp3 was confirmed by Southern blot analysis.

**Table 2:** Oligonucleotides used in this study.

Description	Sequence*
FlankA1	AAA**GGATCC**GCTGTCGTCATCAGAGGGCTGTC
FlankA2	GGTCAAAATGTGGTCAAAAATCAATTTTAAACAAAAAACAAGC
FlankB1	AAATTGATTTTTGACCACTTTTTGACCTTCTTTGTAGTATCTTTTC
FlankB2	TTTT**CCATGG**CAGCAGCGGAAACGCATCCTTGC
testA	TCGATGTGTGACCGAGACGCGTAC
testB	CGAGTGACGACGAAGTTTCC

*Restriction sites within the oligonucleotide sequences are printed in bold.

### Phage particle isolation

*B*. *licheniformis* overnight cultures were used to inoculate 4 ml LB in a test tube with a starting OD_600_ of 0.05. The cultures were grown at 37°C with vigorous shaking. Prophages were induced 2 hours after inoculation using mitomycin C (Sigma-Aldrich, St. Louis, USA) as induction agent with a final concentration of 0.5 μg/ml. A non-induced culture was used as control and cell growth was followed by optical density measurement (Klett-Summerson Photoelectric Colorimeter with a green filter; KLETT Mfg. Co. Inc., NY). Eight hours after induction the supernatant was separated from the cells via centrifugation at 2,500 g for 5 min, followed by sterile filtration with a 0.45μm pore size filter (Sarstedt, Nümbrecht, Germany). Lysozyme from chicken egg white (10 μg/ml, SERVA Heidelberg, Germany) was added to the filtrated supernatant to disrupt the cell walls of potentially remaining host cells. RNase A (Qiagen, Hilden, Germany) and DNase I (Roche Diagnostics, Mannheim, Germany) were added to a final concentration of 10 μg/ml each to the filtrate. The mixture was incubated overnight (16 h) at 37°C. After the enzymatic removal of free nucleic acids, the phage particles were sedimented by ultracentrifugation using a Sorvall Ultracentrifuge OTD50B with a 60Ti rotor applying 200,000 g for 4 h. The supernatant was discarded and the pellet was solved in 200 μl TMK buffer [24], and stored at 4°C or directly used for DNA isolation.

### Phage DNA isolation

The DNA isolation was performed using a MasterPure DNA Purification Kit from Epicentre (Madison, WI, USA). 200 μl 2x T&C-Lysis solution containing 1 μl Proteinase K was added to 200 μl of the phage suspensions and incubated for 15 min at 65°C. After addition of 200 μl MPC solution, proteins were precipitated by centrifugation for 10 min at 10,000 g. The supernatant was transferred to a new tube, mixed with 670 μl cold isopropanol and incubated for 10 min at −20°C. DNA precipitation was performed by centrifugation for 10 min at 17,000 g and 4°C. The DNA pellet was washed twice with 150 μl 75% Ethanol, air-dried and re-suspended in DNase free water.

### DNase I treatment control

To test the efficiency of the enzymatic removal of free nucleic acids in the phage suspension, we performed a control experiment using conditions comparable to the DNase I treatment of the phage particle isolation protocol. Different chromosomal DNA dilutions in LB medium (16 μg/ml, 8 μg/ml, 4 μg/ml, 2 μg/ml and 1 μg/ml) were incubated for 16 h at 37°C with and without 10 μg/ml DNase I (Roche Diagnostics, Mannheim, Germany). To test the efficiency of the treatment, 1 μl of each dilution was used as template for a PCR reaction with the primer pair testA/testB and the Phusion High-Fidelity DNA Polymerase (Thermo Scientific, Darmstadt, Germany), according to the recommendations of the supplier. The control PCRs are constructed to generate products with a size of 159 bp from chromosomal DNA.

### Next generation sequencing (NGS) and data processing

NGS phage DNA libraries were generated with the NEBNext ultra DNA kit (NEB, Ipswich, MA, USA) and Nextera XT DNA Sample Preparation Kit (Illumina, San Diego, USA), and the sequencing was performed on an Illumina GAii sequencer (Illumina, San Diego, USA). The generated sequence reads (Table 3) were mapped on the genome of *B*. *licheniformis* DSM13 (accession number AE017333.1) [10] using bowtie2 [25], allowing 2% aberration. The output SAM files were converted to tds-files by the program samtotds and visualized by TraV [26]. The sequencing raw data are available at the NIH short read archive (SRP035551). To compare phage DNA mappings with different read numbers, a coverage analysis was performed using NPKM (**n**ucleotide activity **p**er **k**ilobase of exon model per **m**illion mapped reads) values [10,26]. NPKM values are normalized mapping values, calculated from the coverage of a defined region in relation to the overall genome coverage. In this paper, activity is defined as the abundance of phage DNA reads, generated from phage particle derived DNA, which correlates to the phage activity. For showing the hotspots of phage activity, NPKM values were calculated for continuous 1 kb-segments over the whole *B*. *licheniformis* DSM13 genome by TraV [26] and used to draw phage activity graphs in MS-Excel.

**Table 3:** NGS read coverage of phage DNA preparations from *B*. *licheniformis* strains.

Strain for phage DNA preparation	Mapped reads	Unmapped reads
*B*. *licheniformis* DSM13	1,278,534	24,221
*B*. *licheniformis* MW3	3,195,030	41,897
*B*. *licheniformis* ΔPBSX	3,515,890	40,356
*B*. *licheniformis* ΔPBSX-ΔBLi_Pp3 (1. exp.)	5,384,670	1,788,078
*B*. *licheniformis* ΔPBSX-ΔBLi_Pp3 (2. exp.)	194,173	94,872
*B*. *licheniformis* ΔPBSX-ΔBLi_Pp3 (3. exp.)	2,030,737	53,687

The sequencing raw data are available at the NCBI short read archive (SRP035551).

### Signal to noise ratio determination

The signal to noise ratios for the phage DNA mappings were calculated from base coverage determined by TraV [26]. The base coverage is defined as the number of reads mapped over a specific base during the mapping process. The average base coverage in the prophage regions (signal) and the nonprophage regions (noise) were calculated by counting the number of reads and dividing them by the respective region size. The signal to noise ratios were calculated by dividing the signal values by the noise values. In addition, the relative noise (%) in relation to the signal was calculated by setting the signal values to 100%.

### Negative staining and transmission electron microscopy

For negative staining, a carbon film, evaporated on a freshly cleaved mica surface, was partially floated off the mica by dipping into a drop of phage particle suspension. The carbon-mica sandwich with the adsorbed biological material was then transferred to a drop of washing solution (deionized water) and then completely floated off on a drop of negative staining solution, where it was transferred onto a 400 mesh specimen grid. The staining solution was completely removed, resulting in a shallowly stained specimen [27,28]. Electron micrographs of phage particles were taken at calibrated magnifications with a JEM 1011 transmission electron microscope (Jeol Ltd., Eching, Germany). Images were taken with an Orius SC 1000 A CCD camera and processed with the digital micrograph image processing software (Gatan, Munich, Germany).

### Protein comparison of prophage regions BLi_Pp1 – BLi_Pp7

The proteins of each prophage region were compared to the remaining six regions with a focus on putative phage repressor proteins. Therefore, the BLi_Pp1 – BLi_Pp7 protein sequences were extracted from the *B*. *licheniformis* DSM13 genbank file (accession number NC_006322.1) by the script cds_extractor (v0.6) (https://github.com/aleimba/bac-genomics-scripts). A BLASTP comparison was performed for every prophage region against a protein database comprising the protein sequences of the remaining six prophage regions. Subsequently, the resulting protein pairs were aligned using the Needleman-Wunsch-Algorithm [29]. The annotations of the prophage regions were searched for known and putative repressors and the generated Needleman-Wunsch scores were used for the comparison of BLi_Pp1 – BLi_Pp7 (S1 Table), putative repressor proteins and related regulon proteins were marked in blue).

### Phylogenetic classification of prophage regions

Phylogenetic classification of the identified prophage regions was done by comparison to known phages. In total, 979 bacterial phage genomes and the associated taxonomic information (Table 4) from NCBI’s viral genome page were used (http://www.ncbi.nlm.nih.gov/genomes, viruses; data taken on June 5th 2013). A BLAST phage protein sequence database was created comprising 91,518 phage protein sequences, extracted from the genbank format-files by the script cds_extractor (v0.6) (https://github.com/aleimba/bac-genomics-scripts) and supplemented by the protein sequences of *B*. *subtilis* 168 PBSX phage (NC_000964). BLASTP comparisons were performed for all *B*. *licheniformis* phage proteins to the phage protein database. Subsequently, all hits were aligned by the needle program of EMBOSS suite [29] with default values. By comparing the similarity scores of all pairs with full-length alignment the best hit for every phage protein was determined (S2 Table). Hits with similarity < 30% were discarded.

**Table 4:** Distribution of bacterial phage reference genomes used for the phylogenetic classification.

Phage family	Genetic material	Number of genomes
*Microviridae*	*ssDNA phages*	16
*Inoviridae*		32
*Corticoviridae*	*dsDNA phages*	1
*Myoviridae*		226
*Plasmaviridae*		1
*Podoviridae*		158
*Siphoviridae*		425
*Tectiviridae*		4
*unclassified*		62
*Cytoviridae*	*dsRNA phages*	5
*Leviviridae*	*ssRNA phages*	11

## Results and Discussion

### Prophage identification

The genome sequence of *B*. *licheniformis* DSM13 was initially scanned with the prediction tool Prophage Finder [15]. The nine predicted prophage regions were evaluated based on a set of genomic features and thus, seven regions were annotated as prophage regions BLi_Pp1 – BLi_Pp7 (access code AE017333.1). The two remaining predicted regions were evaluated as false positives and not considered further. The criteria used for evaluation of the prophage predictions were similarity to known phage genes, GC content deviation and the presence of insertion repeats (Table 5). The final seven genomic loci are covering 4.7% of the complete genome.

**Table 5:** Prophage regions of *B*. *licheniformis* DSM13.

Prophage	GC content*	Size (bp)	Insertion repeats^#^	Location
BLi_Pp1	38.10	11,177	61 −6 bp	927,299–938,595
BLi_Pp2	47.05	27,509	no	1,317,754–1,345,262
BLi_Pp3	42.50	41,566	27 −1 bp	1,422,556–1,464,174
BLi_Pp4	44.73	38,319	256 −13 bp	1,504,028–1,542,847
BLi_Pp5	39.89	10,524	50 −3 bp	2,855,587–2,866,209
BLi_Pp6	40.95	44,793	18 bp	3,424,376–3,469,186
BLi_Pp7	35.58	21,733	19 −1 bp	4,155,490–4,177,258

*The average host genome GC content is 46.2. The size of the prophage regions refers to the sequence between the insertion repeats. For BLi_Pp2 the range from the first to the last identified phage gene was used since no insertion repeats were found.

^#^ Size of the insertion repeats with the number of variations in bp.

The regions BLi_Pp1, BLi_Pp5 and BLi_Pp7 are encoded by genome loci of a size between 11 kb and 22 kb. In all three regions open reading frames (ORFs) with similarity to phage-related genes could be identified. An evaluation of the GC content of the prophage regions indicates a clear GC content deviation compared to the host genome (Table 5). Furthermore, the regions are flanked by insertion repeats. The region BLi_Pp1 is located next to a tRNA cluster which is similar to known phage integration sites [30,31]. A genome comparison with *Bacillus licheniformis* 9945A [21] and *Bacillus subtilis* 168 [22,32] revealed the absence of orthologous prophage regions in these strains, indicating that BLi_Pp1, BLi_Pp5 and BLi_Pp7 are part of the DSM13 accessory genome. The region BLi_Pp7 contains the genes of a restriction modification system, which has been reported as a gene class also identified in other prophages [33,34].

BLi_Pp3, BLi_Pp4 and BLi_Pp6 are prophage regions with a size between 38 kb and 45 kb. The GC contents of BLi_Pp3 and BLi_Pp6 also deviate from the host genome and all three loci are flanked by insertion repeats (Table 5). Again, no orthologous prophage regions could be found in *B*. *licheniformis* 9945A and *B*. *subtilis* 168.

Prophage BLi_Pp2 was bioinformatically predicted due to ORFs with similarity to phage genes. The strong similarity to the PBSX prophage locus of *B*. *subtilis* 168 [35] indicates that BLi_Pp2 represents a *B*. *licheniformis* orthologue of the PBSX prophage. For BLi_Pp2 no flanking insertion repeats could be identified and according to the GC-content, no clear sequence deviation was found (Table 5). However, this is in accordance with the PBSX insertion site in *B*. *subtilis*, where the genomic locus also shows no GC content deviation and no insertion repeats are present.

### Comparison of BLi_Pp1 – BLi_Pp7

The similarity of the seven prophage regions to each other was investigated with a focus on putative phage repressor proteins. These proteins repress the induction of phages to remain the lysogenic state [36,37]. Repressor proteins can also confer immunity to the host strain against superinfection [38]. The annotations of the prophage regions were searched for known and putative repressors. The proteins of each prophage region were compared by BLASTP to the remaining six regions, and a global alignment of the resulting protein pairs was performed using the Needleman-Wunsch-Algorithm [29]. The resulting Needleman-Wunsch scores were used for the comparison of BLi_Pp1 – BLi_Pp7 (S1 Table).

The overall similarity between the prophages is low. BLi_Pp7 does not possess any similarity at all to the remaining six prophage regions (S1 Table). For BLi_Pp1 and BLi_Pp5 significant similarities (scores >30) were only observed for a few proteins (e.g. transposases/integrases, some hypothetical proteins and a terminase large subunit). No repressor proteins could be identified for BLi_Pp1, BLi_Pp5 and BLi_Pp7.

The similarities observed for the prophage regions BLi_Pp2, BLi_Pp3, BLi_Pp4 and BLi_Pp6 are more distinct. The comparison of BLi_Pp2 to BLi_Pp3 shows homology for the proteins BLi01317 – Bli01325. This BLi_Pp2 region comprises the transcriptional repressor Xre and its regulon (XkdB-XkdC-XkdD-XtrA; S1 Table, marked in blue), which has been shown to control the induction of the PBSX phage in *B*. *subtilis* [37,39]. The respective open reading frames (ORFs) in BLi_Pp3 are the repressor YqaE (BLi01433) and parts of its regulon (BLi01444, BLi01443 and BLi01445, regulon proteins are marked in blue in S2 Table). YqaE (syn. SknR) was described as the repressor of the skin element, a prophage-like element of *B*. *subtilis* and belongs also to the Xre family of transcription repressors [40]. BLi_Pp4 and BLi_Pp6 do not possess any homology to the repressors of BLi_Pp2 or BLi_Pp3 and their regulon proteins. Only BLi03634 of prophage BLi_Pp6 is similar to the toxin YqaH of BLi_Pp3 (BLi01438, similarity score 78.3), which is in *B*. *subtilis* encoded by the skin element and executes its lethal effect by inhibition of host DNA replication [40]. Vice versa, BLi_Pp6 encodes only a putative HTH-type transcriptional repressor protein, which shows no homology to the remaining prophage region proteins. For BLi_Pp4 only an HTH-type DNA-binding protein was found, possessing no homology to the remaining prophage regions.

Interestingly, the highest similarity scores between BLi_Pp2 and BLi_Pp3 as well as BLi_Pp6 were observed for the holin-like protein XhlB and the membrane-associated protein XhlA (83% to 90% similarity, marked in green in S1 Table), which are essential for host cell lysis in the *B*. *subtilis* PBSX phage [41]. The associated endolysin XlyA, which is only found in BLi_Pp6, is not essential for functionality [41] and might have been replaced by a different endolysin in BLi_Pp3. BLi_Pp4 seems to encode a different type of host lysis system (BLi01567-BLi01569), for which no or only low similarity proteins could be identified in BLi_Pp2, BLi_Pp3 and BLi_Pp6.

### Prophage classification

To classify the *B*. *licheniformis* DSM13 prophage regions, a comparison to publicly available phage genomes was performed. Initially a database comprising 979 bacterial phage genomes available at the NCBI database was constructed (Table 4). The protein sets encoded by the seven annotated prophage-like regions were compared by BLASTP with this database, followed by a global alignment of the resulting protein pairs using the Needleman-Wunsch-Algorithm [42]. For evaluation of similarity of prophage proteins, the best Needleman-Wunsch alignment score was considered, setting a cut off ≥ 30% for the full-length alignments (S2 Table). 89% of all prophage proteins encoded by region BLi_Pp2 showed top similarities to the proteins encoded by the *B*. *subtilis* prophage PBSX. In total, only four proteins exhibited the top similarity to non-PBSX phage proteins. The proteins of the remaining prophage regions showed similarities to members of the *Caudovirales*. Phages of this order harbor dsDNA within their phage particles [43]. An in-depth evaluation of protein homologies indicated that the majority of the investigated protein sequences match proteins encoded by members of the *Siphoviridae* phage family. However, an assignment of a more in depth taxonomic level was not possible due to the partly incomplete classification of the database hits and the comparably small database size (considering the huge pool of existing phages). Moreover, the mosaic nature of phage genomes [44] makes an unambiguous classification at sequence level very difficult. For example around 54% of the BLi_Pp6 proteins are homologous to the lambda-like *Bacillus* phage phi105 including 26% with top homologies (S2 Table, average similarity score 77), but the remaining BLi_Pp6 proteins are not similar at all to this phage.

Taken together, the prophage regions may be divided into prophages (BLi_Pp2, BLi_Pp3, BLi_Pp4 and BLi_Pp6) and prophage remnants (BLi_Pp1, BLi_P5). BLi_Pp1 and BLi_Pp5 with about 11 kb are probably too small to contain a complete genome of a *Siphoviradae* or *Myoviradae* phage [45,46]. The region BLi_Pp7 shows like BLi_Pp1 and BLi_Pp5 limited homology to known phage genes (S2 Table). However, BLi_Pp7 may have a sufficient size for a complete phage genome. The fact that no phage repressor or repressor-like proteins were identified in BLi_Pp1, BLi_P5 and BLi_Pp7 could be additional evidence that these regions are prophage remnants consisting of harmless or beneficial genes as proposed by Lawrence et al., [3].

### Phage particles and their genetic sources

To check if the identified prophage regions are able to generate phage particles, induction experiments with mitomycin C were performed using *B*. *licheniformis* DSM13 and different deletion mutants (Table 1). After induction, phage particles were purified, and isolated phage DNA was sequenced using NGS. The lab strain *B*. *licheniformis* MW3, a restriction-modification system-negative derivate of *B*. *licheniformis* DSM13, was also used in all experiments since the prophage deletion mutants were constructed in the MW3 background. The behavior of *B*. *licheniformis* DSM13 and MW3 was comparable during the experiments (for details see S1, S2 and S3 Figs.). To exclude a specific contamination with chromosomal host DNA, which would generate mappable reads, the supernatant of the induced *B*. *licheniformis* cultures was treated with lysozyme, DNase and RNase before the phage DNA was liberated from the particles. The efficiency of this procedure was tested in control experiments with different concentrations of chromosomal DNA. The control-PCRs were negative for all enzyme-treated samples, but generated a PCR product for all samples without nuclease treatment (S4 Fig.).

#### Activity of the PBSX-like phage region BLi_Pp2

**Fig 1:**
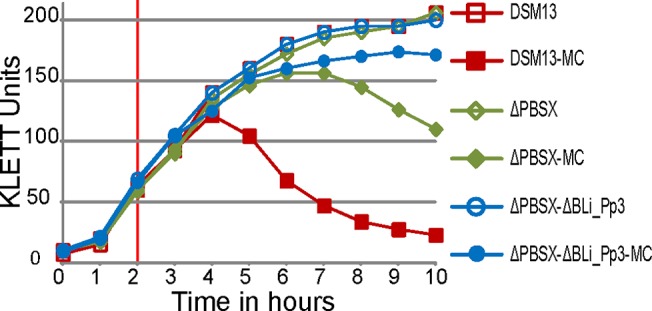
Growth behavior of *B*. *licheniformis* DSM13 and its derivatives after induction with mitomycin C. The KLETT Units represent the turbidity of the culture. The vertical red line at 2 hours marks the time point of induction with 0.5 μg/ml mitomycin C. Induced cultures (MC) are marked with filled symbols and non-induced cultures with open symbols. *B*. *licheniformis* DSM13 shows a loss in turbidity 3 hours after induction with mitomycin C and *B*. *licheniformis* ΔPBSX after 6 hours. The strain *B*. *licheniformis* ΔPBSX-ΔBLi_Pp3 exhibited no decrease of turbidity after mitomycin C treatment.

**Fig 2:**
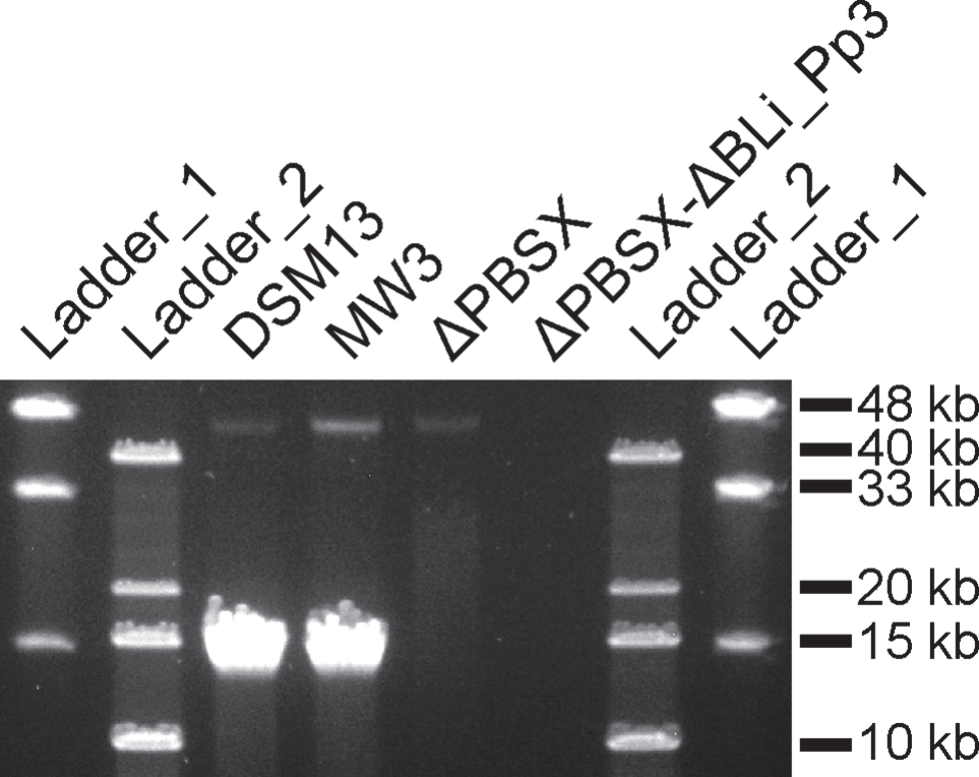
Pulsed field gel electrophoresis (PFGE) of phage DNA isolated from mitomycin C treated *B*. *licheniformis* DSM13 and its derivatives. The 1% gel was run for 18 h at 14°C using a voltage of 6V/cm and switch times ramped from 0.1–10 sec. The phage DNA preparations of *B*. *licheniformis* DSM13 and *B*. *licheniformis* MW3 show a strong band of approximately 13 kb and a weak band of approximately 44 kb. The phage DNA preparation of *B*. *licheniformis* ΔPBSX shows a weak band of approximately 44 kb, and for *B*. *licheniformis* ΔPBSX-ΔBLi_Pp3 no bands could be detected. NEB MidRange I PFG marker (ladder 1) and Invitrogen 1 kb DNA Extension Ladder (ladder 2) were used. The depicted figure has been cropped to improve clarity. S2 Fig. shows a full size gel picture. Uninduced cultures of *B*. *licheniformis* DSM13 and ΔPBSX do not show any DNA bands (data not shown).

**Fig 3:**
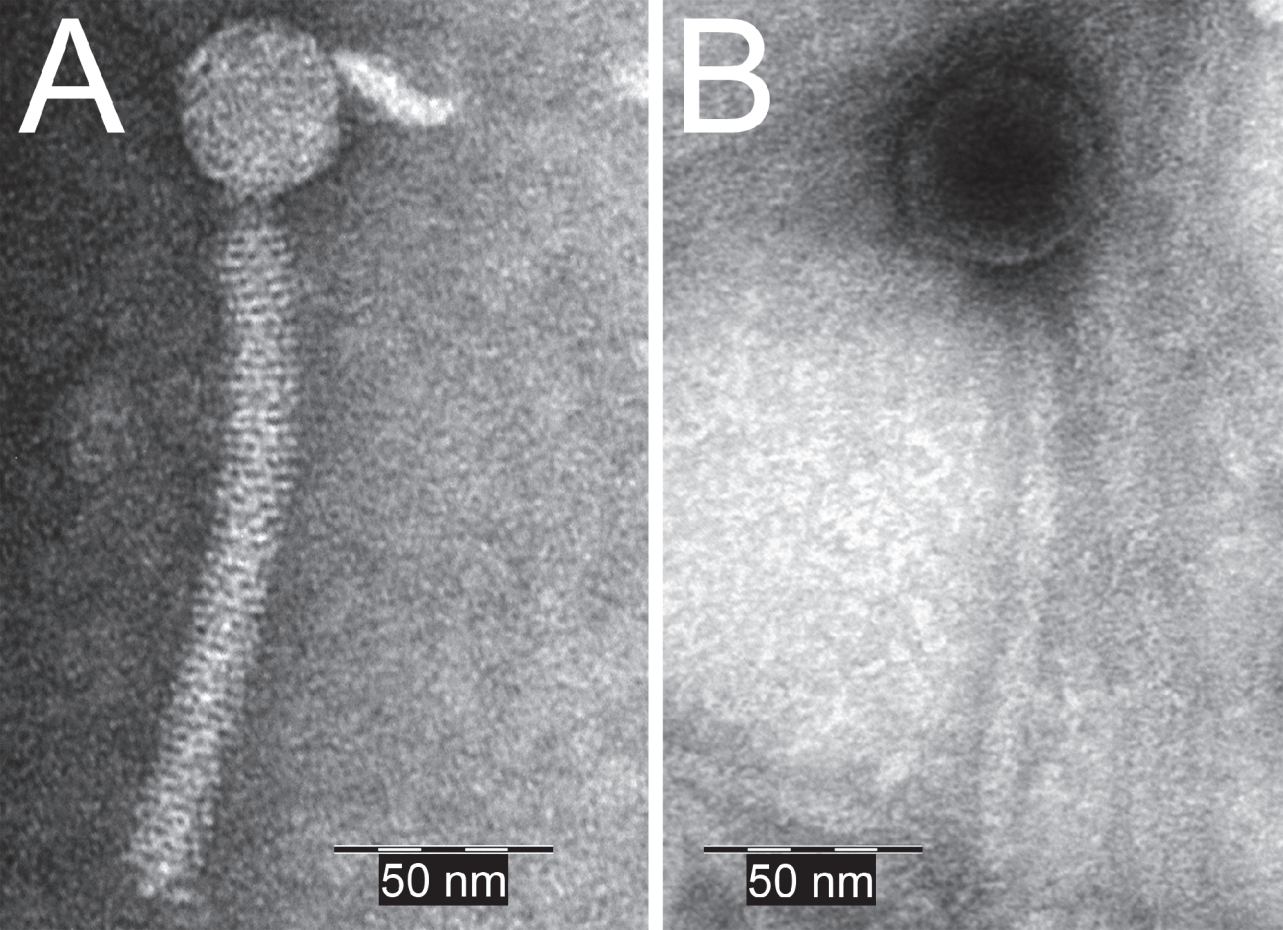
TEM micrographs of negatively stained phage particles detected in *B*. *licheniformis* strains after induction with mitomycin C. A: PBSX-like phage particle, detected in the phage suspension isolated from *B*. *licheniformis* DSM13. B: *Siphoviridae*-like phage particle, detected in the phage suspension of *B*. *licheniformis* ΔPBSX.

**Fig 4:**
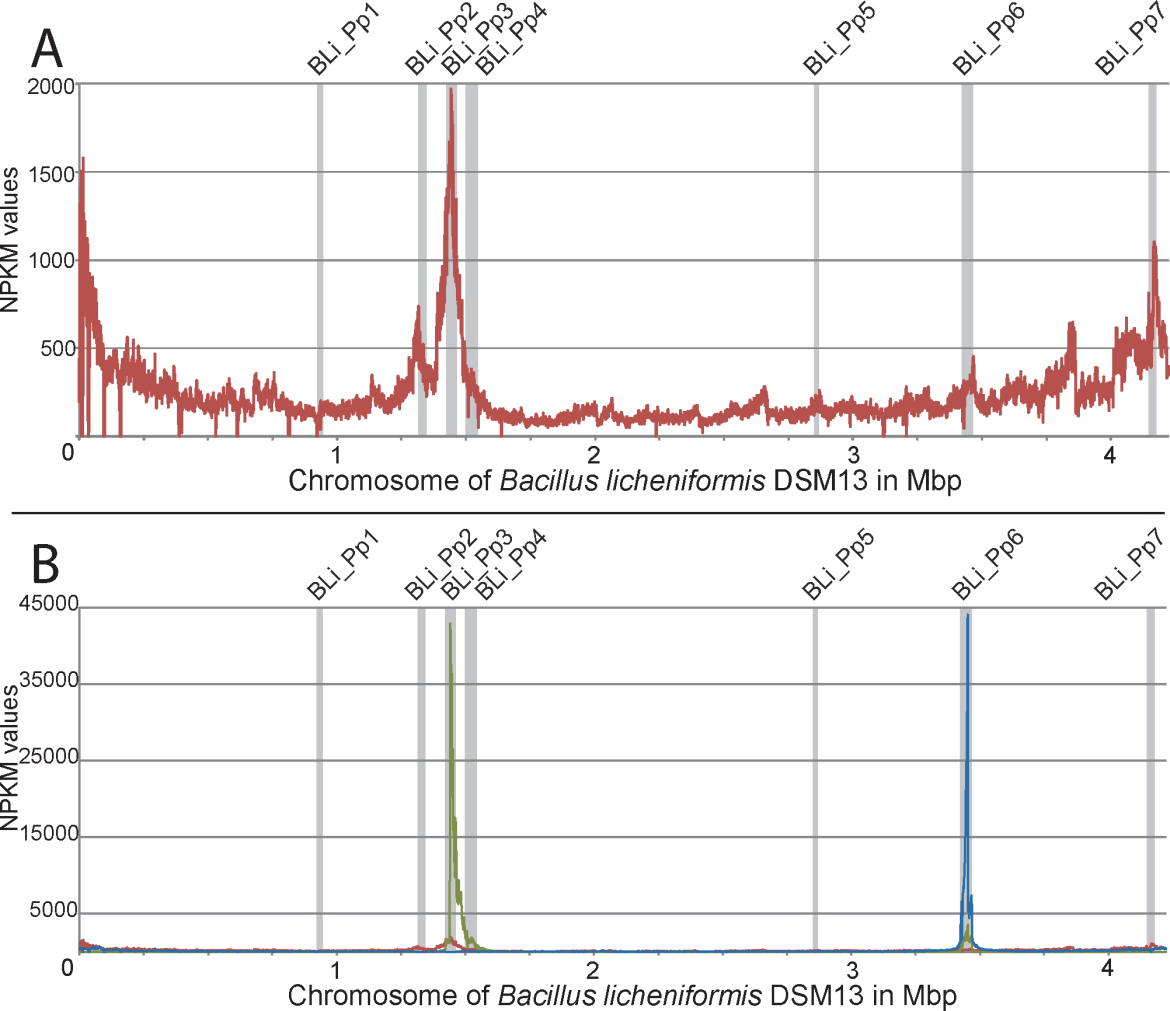
Phage activity graphs on the genome of *B*. *licheniformis* DSM13. *B*. *licheniformis* phage DNA was sequenced by next generation sequencing (NGS) and sequences were mapped on the genome of *B*. *licheniformis* DSM13. The results are displayed in NPKM values calculated by TraV [26]. Prophage regions BLi_Pp1 – BLi_Pp7 are marked with grey bars. A: The phage DNA read mapping of *B*. *licheniformis* DSM13 shows a even distribution of reads over the whole genome as well as read accumulations around the origin of replication and also in some further regions, especially in BLi_Pp3. B: The *B*. *licheniformis* ΔPBSX phage DNA read mapping (green graph) shows a strong read accumulation in the region BLi_Pp3 compared to the *B*. *licheniformis* DSM13 read mapping (red graph), and a slight read accumulation in region BLi_Pp6. The *B*. *licheniformis* ΔPBSX-ΔBLi_Pp3 phage DNA read mapping (blue graph, result of the first experiment is shown) exhibits a distinct read accumulation in BLi_Pp6.

The wild-type strain *B*. *licheniformis* DSM13 showed a clear decrease in turbidity 3 hours after induction (Fig. 1, red graph with filled symbols), which indicates bacterial lysis. Gel electrophoresis of phage DNA purified from the phage suspension showed two DNA bands with a size of approximately 13 kb and 44 kb (Fig. 2). In addition, the TEM-based investigation revealed a type of phage particles, which corresponds in size and appearance to PBSX-like phages (Fig. 3A). The DNA was sequenced by next generation sequencing (NGS) technology and mapped on the genome of *B*. *licheniformis* DSM13. Normalized activity values (NPKM values, for details see method section and [10,26]) were calculated from the read coverage and used to compare the phage DNA mappings from different experiments with prophage deletion mutants and the DSM13 wild type (Fig. 4). The mapping of the *B*. *licheniformis* DSM13 derived phage DNA shows a distribution of the reads over the whole genome (Fig. 4A). The coverage of the prophage regions is with a signal to noise ratio of 2.6 (Table 6) only 2.6 times increased. The relative noise over the whole genome is with 38% (Table 6), strongly increased. The general distribution of reads over the whole genome indicates random packing of chromosomal DNA and supports the assumption of a PBSX ortholog prophage in *B*. *licheniformis* DSM13, since PBSX-like prophages are described to randomly pack 13 kb fragments of the host chromosome instead of their own genome with a size of about 30 kb [24,35,47,48]. The comparison of the results from strains *B*. *licheniformis* DSM13 and MW3 with *B*. *licheniformis* ΔPBSX, a BLi_Pp2 deletion mutant, strongly indicates that prophage BLi_Pp2 is responsible for the huge amount of prophage-unspecific sequence reads. The higher read coverage close to the origin of replication of the *B*. *licheniformis* DSM13 genome (Fig. 4A) can be explained through the higher available amount of DNA of this multi-copy genomic region due to the replication start site [49]. The strongly increased read coverage observed at the BLi_Pp3 region (Fig. 4A) indicates this region as possible origin of the second phage DNA band of approximately 44 kb (Fig. 2). In addition, the prophage regions BLi_Pp2, BLi_Pp5, BLi_Pp6 and BLi_Pp7 exhibit a small read accumulation as well as a non-prophage region at 3.8 Mb. Considering the increased average coverage due to the genomic DNA from the PBSX orthologous phage, no further conclusions can be drawn on this observation at the moment.

**Table 6:** Results of the signal to noise ratio determination.

	DSM13	MW3	ΔPBSX	ΔPBSX ΔBLi_Pp3 (1. exp.)	ΔPBSX ΔBLi_Pp3 (2. exp.)	ΔPBSX ΔBLi_Pp3 (3. exp.)
Average base coverage in prophage regions (signal)	82.7	187	1447.2	1671.8	44.9	664.6
Average base coverage in non-prophage regions (noise)	31.5	79.7	27.2	68.3	1.4	5.4
Signal-to-noise ratio	2.6	2.3	53.2	24.5	32.1	123.1
Noise (%)	38.1%	42.6%	1.9%	4.1%	3.1%	0.8%

The table shows the average base coverage and signal to noise ratios for mappings of NGS experiments of phage DNA isolated from *B*. *licheniformis* DSM13 and MW3, and the mutant strains *B*. *licheniformis* ΔPBSX and ΔPBSX_ΔBLi_Pp3. The average base coverage in prophage regions (signal) and in non-prophage regions (noise) were calculated by counting the number of reads and dividing them by the respective region size. The signal to noise ratio (signal divided by noise) was calculated to compare experiments with different reads numbers. The relative noise (%) gives a percent value for the noise in relation to the signal (set 100%).

The coverage gaps, which can be observed over the whole genome (Fig. 4A), correspond to repetitive regions. The applied mapping method considers only reads, which can be exclusively assigned to a single position within the genome. In addition, the *B*. *licheniformis* MW3 phage DNA mapping (S3 Fig.) exhibits an expected coverage gap at the BLi_Pp7 region, which corresponds to a deletion of the RM system described in [17].

#### Phage activity in the ΔPBSX mutant

A deletion mutant of the PBSX-orthologous region BLi_Pp2, designated as *B*. *licheniformis* ΔPBSX [18], was used in further induction experiments. Six hours after induction with mitomycin C the culture showed first lysis effects (Fig. 1). Gel electrophoresis of the isolated phage DNA revealed an exclusive DNA band of approximately 44 kb (Fig. 2), and TEM investigation of the phage suspension showed the presence of phage particles with a *Siphoviridae*-like morphology (Fig. 3B). In contrast, no phage particles from the PBSX-like particle type (Fig. 3A) could be detected. Thus, we conclude that the particles observed in the induction experiments with *B*. *licheniformis* DSM13 are encoded by the PBSX-like genome locus BLi_Pp2.

The mapping of NGS generated sequences from the second experiment revealed two genomic regions with increased read coverage, one at the locus annotated as BLi_Pp3 reaching into the adjacent downstream genomic region, and the second slight coverage increase at the locus BLi_Pp6 (Fig. 4B, green graph). Unlike the first experiment, no distinct read coverage over the whole genome was observed. The coverage of region BLi_Pp3 is strongly increased and declines for about 200 kb downstream, covering in addition the BLi_Pp4 prophage region. Due to the lack of coverage peaks at the boundaries of the BLi_Pp4 region, it remains unclear whether the locus BLi_Pp4 contributes to the phage particle-derived reads. Consequently, the NGS analysis confirms the activity of at least one more prophage region, seen as a DNA band of approximately 44 kb (Fig. 2) in agarose gel electrophoresis and as phage particle in TEM (Fig. 3B). Due to the high read coverage in the BLi_Pp3 region we expect this prophage to be active.

#### Activity of prophage regions BLi_Pp3 and BLi_Pp6

To confirm this assumption and to elucidate the role of BLi_Pp4, a *B*. *licheniformis* ΔPBSX-ΔBLi_Pp3 double mutant was constructed on the background of *B*. *licheniformis* ΔPBSX [18]. In induction experiments with mitomycin C the double mutant culture exhibited only a slight reduction of turbidity, which might be rather due to the mitomycin C toxicity than to prophage-induced cell lysis (Fig. 1). The phage DNA preparation revealed neither a DNA band on an agarose gel (Fig. 2), nor was any DNA concentration measurable by NanoDrop (Peqlab, Erlangen, Germany). Nevertheless, this DNA preparation generated mappable reads after NGS sequencing (Fig. 4B, blue graph). Strikingly, the experiment showed a clear coverage peak within the BLi_Pp6 region. No phage particles could be observed in the phage suspension of *B*. *licheniformis* ΔPBSX-ΔBLi_Pp3 by TEM, although the BLi_Pp6 DNA might have been protected through a phage particle considering the DNA isolation protocol. The identification of an active prophage locus in the absence of TEM-detectable phage particles and measurable or visible phage DNA highlights the superior sensitivity of NGS based phage identification.

In contrast to BLi_Pp6, the BLi_Pp4 prophage region showed no sequence read accumulation in the *B*. *licheniformis* ΔPBSX-ΔBLi_Pp3 mapping (Fig. 4B). Due to this observation we conclude that BLi_Pp4 does not form functional phage particles or it requires the presence of the genomic locus BLi_Pp3 for full activity. As neither phage particles nor phage DNA could be found in the phage suspension of *B*. *licheniformis* ΔPBSX-ΔBLi_Pp3, we further conclude that the phage particle observed in the single mutant strain *B*. *licheniformis* ΔPBSX (Fig. 3B) and the observed DNA band of approximately 44 kb (Fig. 2) correspond to the BLi_Pp3 prophage region.

Whether BLi_Pp3 has an impact on the expression of BLi_Pp6 is not known. However, BLi_Pp4 and BLi_Pp6 do not show any homology to the repressor regions of BLi_Pp2 and BLi_Pp3, and the overall similarity between the prophage regions is low. The DNA concentration in the phage DNA samples of *B*. *licheniformis* ΔPBSX-ΔBLi_Pp3 was not measurable. Thus, it is more likely that BLi_Pp6 is a low abundant phage and therefore its activity was not clearly visible in experiments with the single mutant strain *B*. *licheniformis* ΔPBSX.

#### Prophage region BLi_Pp7

The evaluation of the prophage region BLi_Pp7 was inconclusive. In *B*. *licheniformis* DSM13 a slight coverage increase for BLi_Pp7 was visible, but this might be due to the overall high read coverage generated by the random packing PBSX-like phage BLi_Pp2. In the *B*. *licheniformis* ΔPBSX mutant and the ΔPBSX-ΔBLi_Pp3 double mutant strains no increased read coverage was visible on the BLi_Pp7 loci after induction with mitomycin C. However, no conclusion on the BLi_Pp7 character can be drawn from the last observations. Since the mutant strains were constructed in the MW3 background, in which the RM system gene *hsdR*2 of the BLi_Pp7 region is deleted [17], the potential prophage locus is not in its native state. Due to the lack of mutants with DSM13 instead of MW3 genomic background, the activity of the BLi_Pp7 region has not been further investigated.

### NGS-based phage identification

The use of NGS for the investigation of phage activity by sequencing of particle-protected phage DNA is a new, sensitive approach. It allows fast investigation of the capability of prophages to form particles and a specific assignment of the activity to a specific genome locus. A recently published investigation of extra-chromosomal bacteriophages [50] also utilized an NGS approach. In this study, after the enrichment of extra-chromosomal DNA, NGS was performed to determine the appearance and state of these elements. Our approach targets particle-forming phages, thus showing their activity and particle content. Similar approaches utilize PCR analysis after induction with mitomycin C to show the presence of specific phage DNAs [50,51], but do not address the whole phage genome.

This study was conducted with the intention to investigate dsDNA phage genomes, and thus, dsDNA specific protocols were used for DNA isolation, library preparation and sequencing. To our knowledge there is no report of *Bacillus* phages that do not pack their genomes as dsDNA. However, there are phages, like members of the family *Inoviridae*, which pack their genomes as ssDNA and also have the ability to lysogenize their hosts [52]. Genome sequences of *Inoviridae*, deposited at the NCBI database, have a size of about 4.5 kb—10.5 kb, similar to the small prophage regions BLi_Pp1 (11 kb) and BLi_Pp5 (10.5 kb). The evaluation of the sequence comparisons does not indicate that the two regions belong to a known ssDNA generating phage class. However, we cannot exclude that BLi_Pp1 and BLi_Pp5 might belong to unknown classes of ssDNA or RNA packing phages and thus, would not have been detected with our methodology.

#### Sequencing of low abundant phages

The sequencing of phage DNA from the double mutant strain *B*. *licheniformis* ΔPBSX-ΔBLi_Pp3 revealed a distinct coverage peak for prophage region BLi_Pp6, although no DNA concentration was measurable or visible for this sample. However, a considerably high portion of unmapped reads was observed during the read mapping (Table 3). Consequently, the induction experiments for this strain where performed in triplicates (exp. 1–3).

For evaluation of the background noise of the read mappings, the relative percentage of noise was calculated (Table 6). The relative noise for *B*. *licheniformis* DSM13 and MW3 is 38.1% and 42.6% in non-prophage regions, respectively. For the *B*. *licheniformis* single mutant strains ΔPBSX, the majority of reads map within prophage regions and only 1.9% relative noise was detected. For the double mutant strain *B*. *licheniformis* ΔPBSX_ΔBLi_Pp3 the relative noises are comparably low for the first two experiments (4-3%) and for the third experiment even <1%. The high signal to noise ratios (24.5–123.1) and the comparable mappings for the *B*. *licheniformis* ΔPBSX_ΔBLi_Pp3 experiments (S5 Fig.) show that the results are unaffected by the number of unmapped reads.

Finally, we would like to point out that in our experiments with the *B*. *licheniformis* DSM13 wild-type strain the presence of the predominant PBSX-like prophage BLi_Pp2 hides the activities of the loci that generate low abundant phage particles. As PBSX-like prophages are distributed in many *Bacillus* strains [13], we expect a similar situation in the whole *B*. *subtilis* species complex. Thus, the application of our workflow, including the deletion of the predominant PBSX-like prophage, might result in the identification of currently unidentified phages.

## Conclusion

Here we demonstrate how genomic methods can be used in the field of prophage analysis. Seven prophage-like regions could be identified in *B*. *licheniformis* DSM13 by comparative genomics, one PBSX-like prophage (BLi_Pp2) and six prophage regions with homology to known members of the *Siphoviridae*. The applied NGS approach confirmed the activity of two prophages from literature and revealed in addition a third active prophage. The read mapping and coverage analysis enabled the assignment of the active prophages to the genome regions BLi_Pp2, BLi_Pp3 and BLi_Pp6. The described workflow combines genomics and classical TEM- and gel-based methods for prophage analysis.

## Supporting Information

S1 Fig Prophage induction in *B*. *licheniformis* DSM13 and MW3. The KLETT Units represent the turbidity of the culture. The vertical red line at 2 hours marks the induction point with 0.5 μg/ml mitomycin C. Induced cultures (MC) are marked with filled symbols and non-induced cultures with open symbols. *B*. *licheniformis* DSM13 and MW3 show a loss in turbidity 3 hours after induction with mitomycin C.
(TIF)

S2 Fig Pulsed field gel electrophoresis of phage DNA isolated from *B*. *licheniformis* DSM13 and its derivatives (full size). The 1% gel was run for 18 h at 14°C using a voltage of 6V/cm and switch times ramped from 0.1–10 sec. The phage DNA preparations of *B*. *licheniformis* DSM13 and *B*. *licheniformis* MW3 show a strong band of approximately 13 kb and a weak band of approximately 44 kb. The phage DNA preparation of *B*. *licheniformis* ΔPBSX shows a weak band of approximately 44 kb, and for *B*. *licheniformis* ΔPBSX-ΔBLi_Pp3 no bands could be detected. NEB MidRange I PFG marker and Invitrogen 1 kb DNA Extension Ladder were used.
(TIF)

S3 Fig Phage activity graphs of *B*. *licheniformis* DSM13 and MW3 on the genome of *B*. *licheniformis* DSM13. After mitomycin C induction *B*. *licheniformis* phage DNA was isolated and sequenced by next generation sequencing (NGS). The sequences were mapped on the genome of *B*. *licheniformis* DSM13 and the NPKM value calculation was performed by TraV [26]. Prophage regions BLi_Pp1 – BLi_Pp7 are marked with grey bars. The read mappings of *B*. *licheniformis* DSM13 (red graph) and *B*. *licheniformis* MW3 (yellow graph) are comparable.
(TIF)

S4 Fig Efficiency test for the DNase I treatment of the phage DNA preparation protocol. Different concentrations of chromosomal DNA were treated with DNase I and afterwards used for PCR. All PCRs with DNase I-treated chromosomal DNA samples did not result in a PCR product. All PCRs with non-treated chromosomal DNA generated an expected 159 bp control fragment. GeneRuler 1 kb Plus DNA Ladder (Thermo Scientific) was used as length standard.
(TIF)

S5 Fig Graphical comparison of three NGS experiments on *B*. *licheniformis* mutant ΔPBSX-ΔBLi_Pp3. Three independent phage DNA preparations were sequenced and mapped to the genome of *B*. *licheniformis* DSM13. The results are displayed in NPKM values calculated by TraV [26]. The three experiments (**A.—C**., exp. 1. -3.) of *B*. *licheniformis* ΔPBSX-ΔBLi_Pp3 are comparable. All three mappings show a clear read accumulation at the BLi_Pp6 prophage region.
(TIF)

S1 Table Annotation of the prophage regions BLi_Pp1 – BLi_Pp7 and results of the BLASTP comparison and Needleman-Wunsch alignment of the seven prophage regions to each other. The resulting Needleman-Wunsch similarity scores are colored according to similarity classes. Scores of 0–25 are not marked, low scores of 25–50 are marked in yellow, medium scores of 50–75 are marked in orange and high scores of 75–100 are marked in red. If more than one hit was found for one query protein the respective higher similarity score was used. Differences in the comparison of two prophage regions, dependent on the BLASTP direction, are due to paralogous proteins.
(XLSX)

S2 Table Protein comparison of the *B*. *licheniformis* DSM13 prophage regions BLi_Pp1—BLi_Pp7 to known phage proteins. (XLSX)
