# Supplementary figures and images for: Genome-Based Identification of Active Prophage Regions by Next Generation Sequencing in Bacillus licheniformis DSM13
Source: PLoS One. 2015 Mar 26;10(3):e0120759. doi: 10.1371/journal.pone.0120759 (PMC4374763; doi:10.1371/journal.pone.0120759)

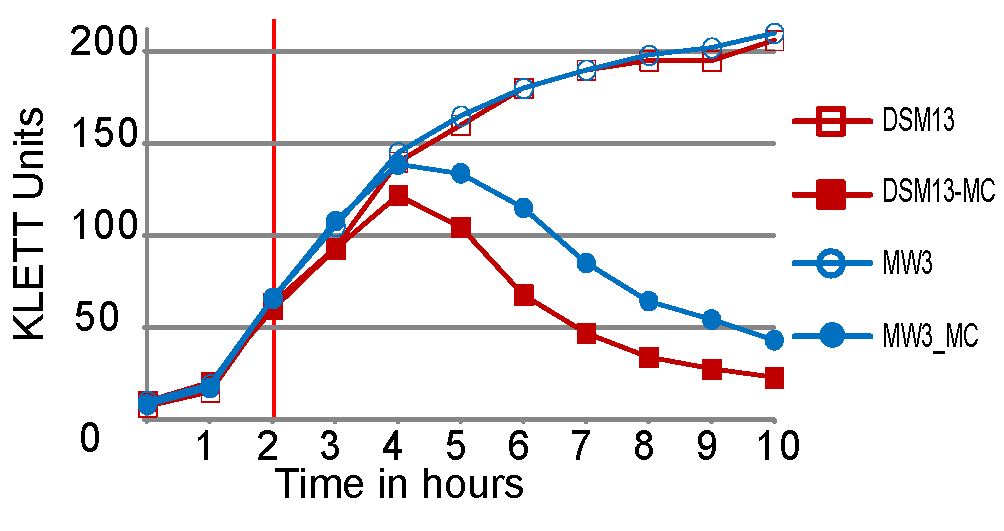

Supplement: S1 Fig — The KLETT Units represent the turbidity of the culture. The vertical red line at 2 hours marks the induction point with 0.5 μg/ml mitomycin C. Induced cultures (MC) are marked with filled symbols and non-induced cultures with open symbols. B. licheniformis DSM13 and MW3 show a loss in turbidity 3 hours after induction with mitomycin C. (TIF) [file pone.0120759.s001.tif]

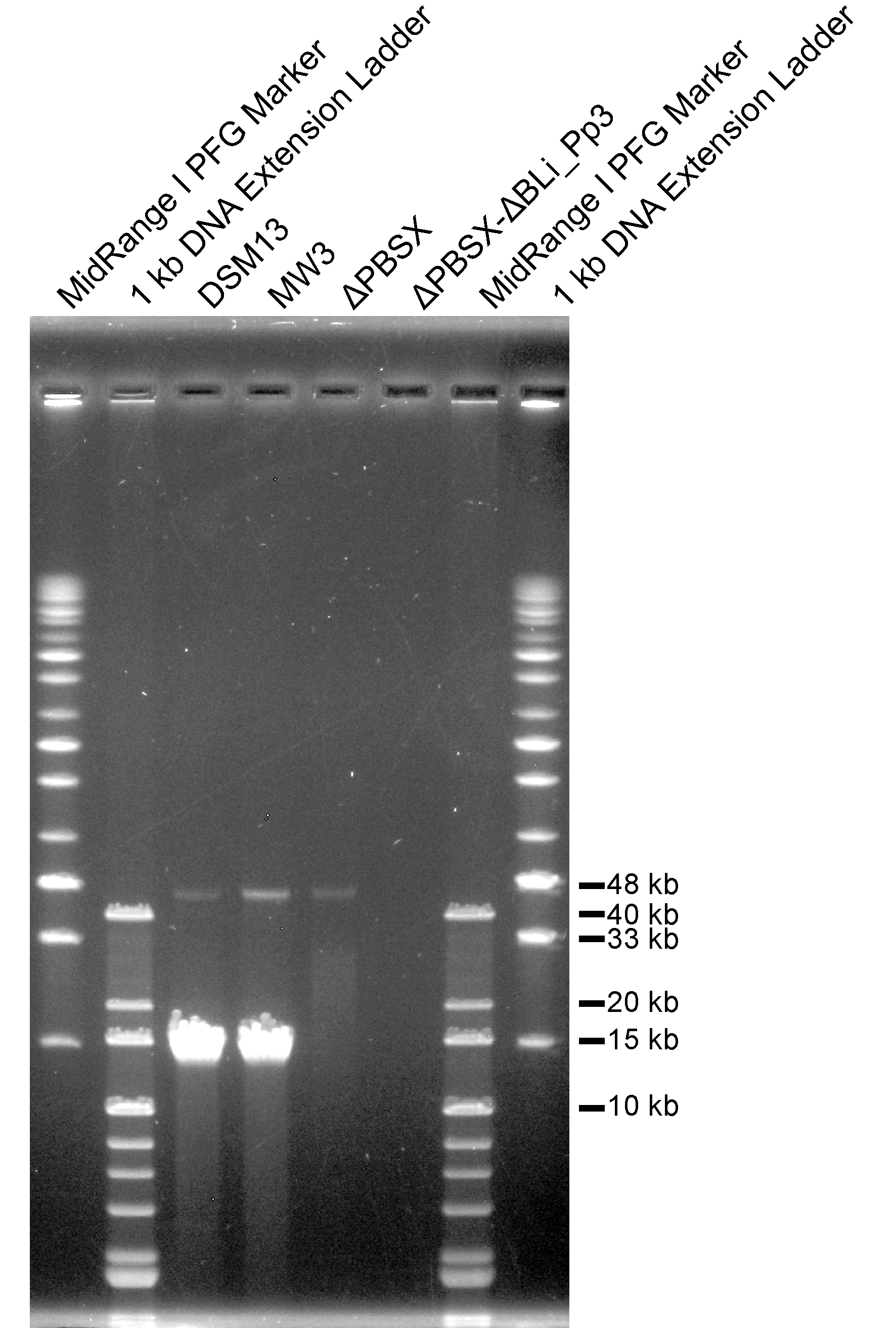

Supplement: S2 Fig — The 1% gel was run for 18 h at 14°C using a voltage of 6V/cm and switch times ramped from 0.1–10 sec. The phage DNA preparations of B. licheniformis DSM13 and B. licheniformis MW3 show a strong band of approximately 13 kb and a weak band of approximately 44 kb. The phage DNA preparation of B. licheniformis ΔPBSX shows a weak band of approximately 44 kb, and for B. licheniformis ΔPBSX-ΔBLi_Pp3 no bands could be detected. NEB MidRange I PFG marker and Invitrogen 1 kb DNA Extension Ladder were used. (TIF) [file pone.0120759.s002.tif]

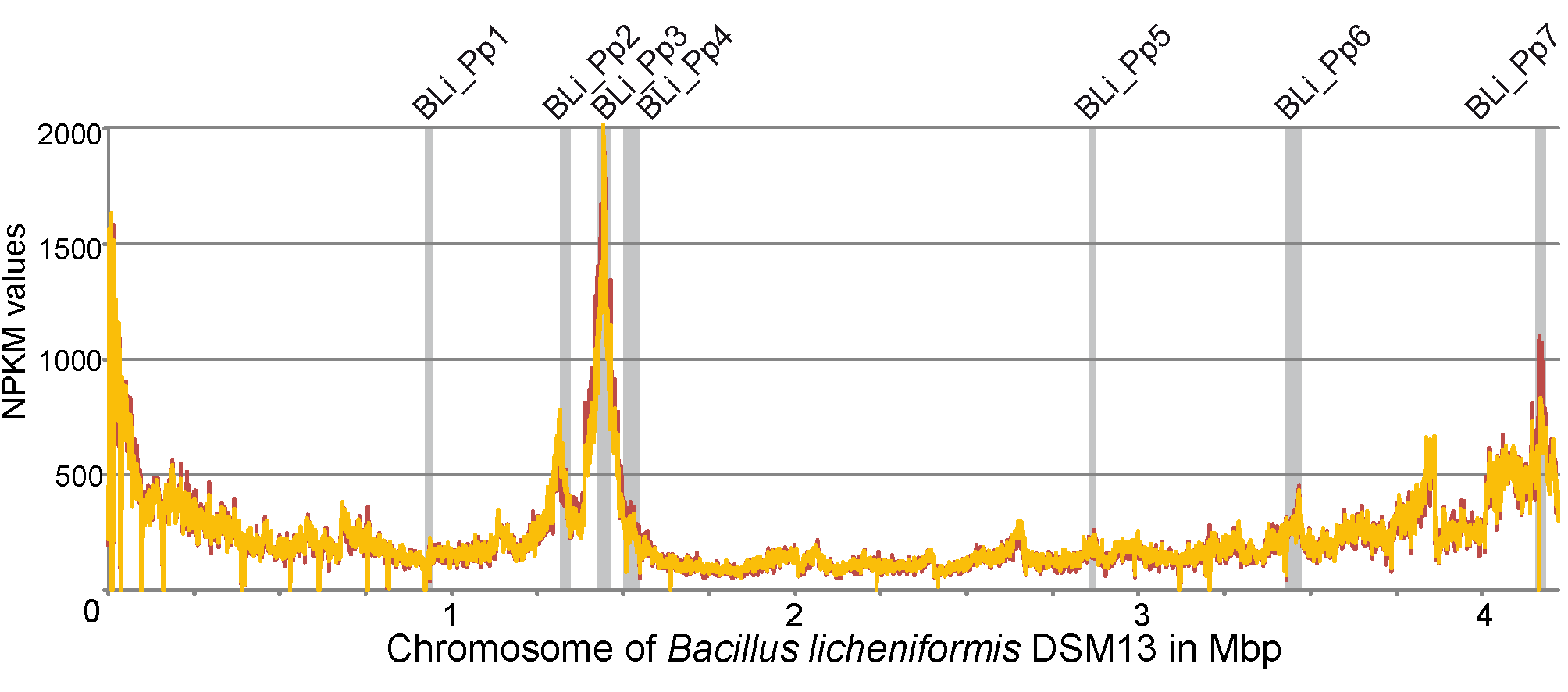

Supplement: S3 Fig — After mitomycin C induction B. licheniformis phage DNA was isolated and sequenced by next generation sequencing (NGS). The sequences were mapped on the genome of B. licheniformis DSM13 and the NPKM value calculation was performed by TraV [26]. Prophage regions BLi_Pp1 – BLi_Pp7 are marked with grey bars. The read mappings of B. licheniformis DSM13 (red graph) and B. licheniformis MW3 (yellow graph) are comparable. (TIF) [file pone.0120759.s003.tif]

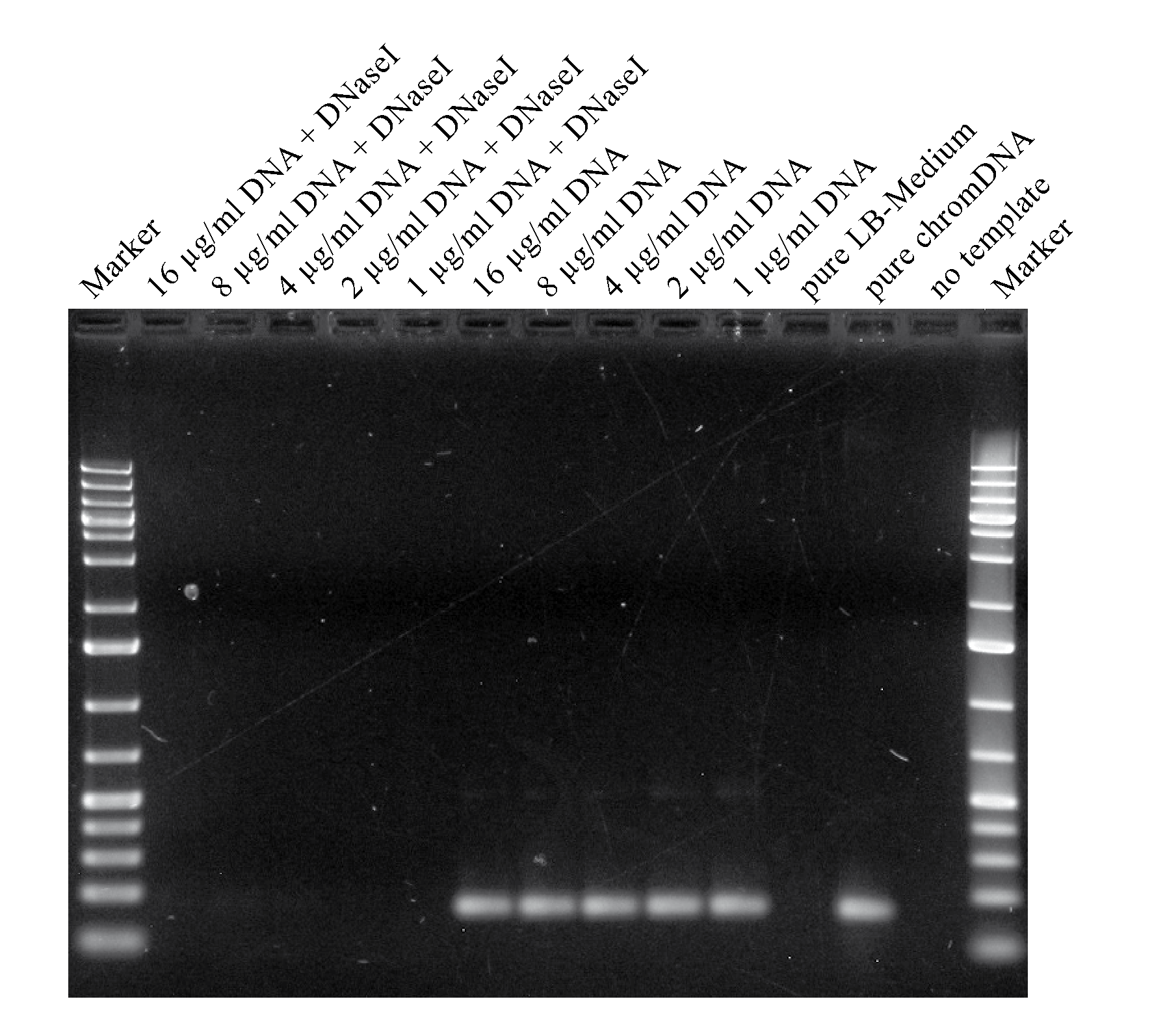

Supplement: S4 Fig — Different concentrations of chromosomal DNA were treated with DNase I and afterwards used for PCR. All PCRs with DNase I-treated chromosomal DNA samples did not result in a PCR product. All PCRs with non-treated chromosomal DNA generated an expected 159 bp control fragment. GeneRuler 1 kb Plus DNA Ladder (Thermo Scientific) was used as length standard. (TIF) [file pone.0120759.s004.tif]

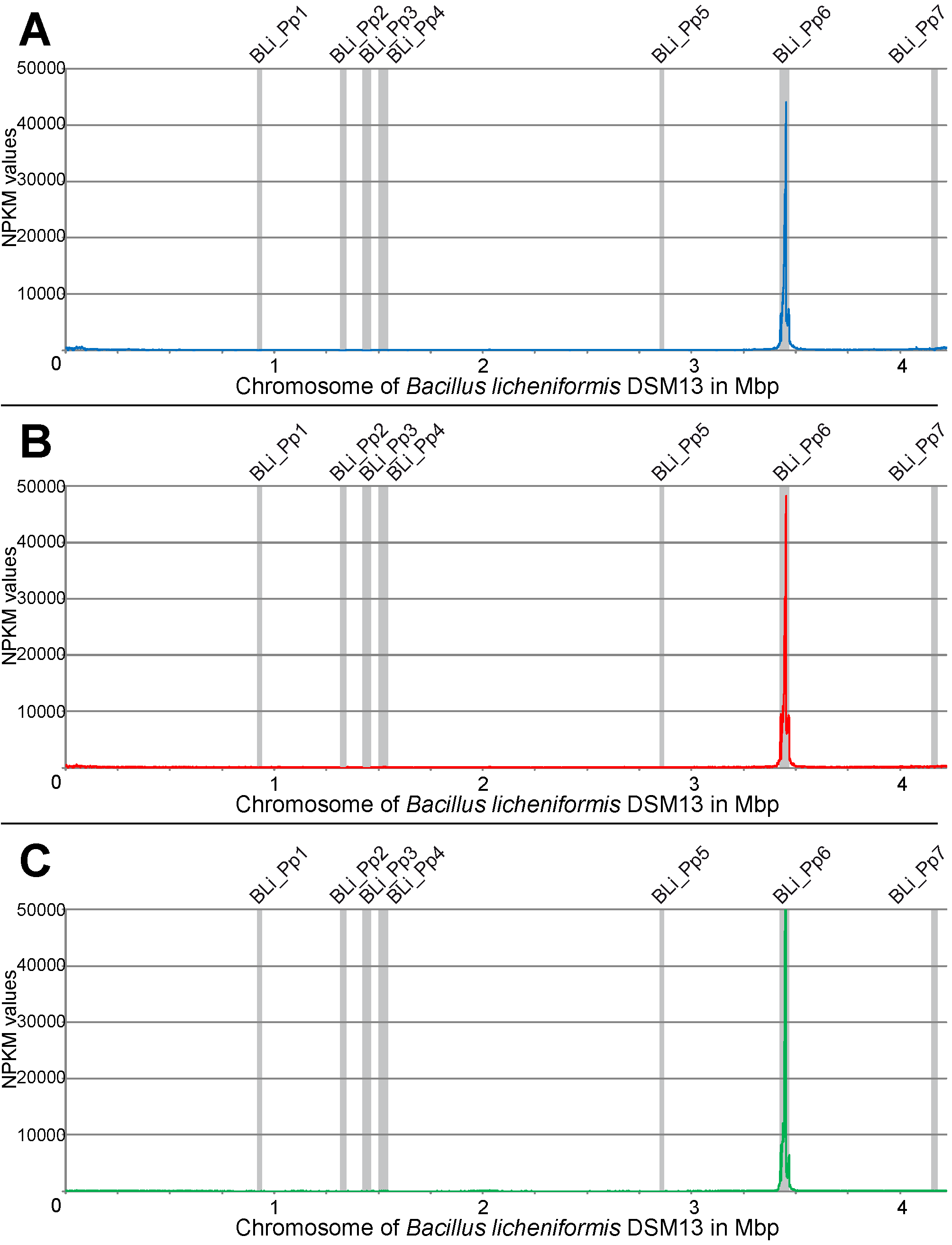

Supplement: S5 Fig — Three independent phage DNA preparations were sequenced and mapped to the genome of B. licheniformis DSM13. The results are displayed in NPKM values calculated by TraV [26]. The three experiments (A.—C., exp. 1. -3.) of B. licheniformis ΔPBSX-ΔBLi_Pp3 are comparable. All three mappings show a clear read accumulation at the BLi_Pp6 prophage region. (TIF) [file pone.0120759.s005.tif]
